# The Caloric Necessities of Critical Care Patients During the First Week of Admission

**DOI:** 10.7759/cureus.33999

**Published:** 2023-01-20

**Authors:** Rita P Medeiros, Ricardo Filipe Ramos de Sousa, Mariana Santos Silva, Rita Rego, Cristina Torrao, Inês M Amaral, Rita Pereira, João P Pinho, Ricardo C Sousa Marinho, Aníbal D Sousa Marinho

**Affiliations:** 1 Intensive Care Unit, Centro Hospitalar Universitário do Porto (CHUP), Porto, PRT; 2 Family Medicine, Unidade de Saúde Familiar (USF) Terras de Souza, Porto, PRT; 3 Nutrition, Associação Portuguesa de Nutrição Entérica e Parentérica, Porto, PRT; 4 Internal Medicine, Centro Hospitalar Universitário do Porto (CHUP), Porto, PRT; 5 Nutrition, Centro Hospitalar do Médio Ave, Vila Nova de Famalicão, PRT

**Keywords:** energy expenditure, nutritional support, indirect, calorimetry, critical illness

## Abstract

Introduction: The nutritional needs of critically ill patients have been the subject of intense controversy. In accordance with international guidelines, it is advocated to optimize a nutritional intake based on the following recommendation: 25-30 kcal/kg body weight per day. However, there still are authors who recommend permissive underfeeding in the first week of hospitalization. Nevertheless, energy expenditure (EE) and necessity are influenced by the catabolic phase of critical illness, which may vary over time on a patient and from patient to patient.

Objective: The objective of this study is to assess if the energy needs of critically ill patients admitted in our intensive care unit (ICU) in the first week of hospitalization are in line with those recommended by the European Society for Clinical Nutrition and Metabolism (ESPEN) international guidelines.

Methods: A prospective cross-sectional study was carried out from September to December 2019. The energy needs were evaluated by indirect calorimetry and by the Harris-Benedict equation. Stress variables were evaluated, namely, the type of pathology, hemodynamic support, sedation, temperature, sequential organ failure assessment (SOFA) score, and state at discharge.

Results: Forty-six patients were included in this study, with an average energy expenditure by indirect calorimetry of 19.22 ± 4.67 kcal/kg/day. The energy expenditure was less than 20 kcal/kg/day in 63% of the measurements. The concordance rate did not show the relationship between the Harris-Benedict equation and the values of indirect calorimetry. Stress variables were analyzed, with the SOFA score as the only variable with values close to statistical significance.

Conclusion: In our ICU, the energy needs of critically ill patients in the first week of hospitalization are lower than the intake recommended by the guidelines.

## Introduction

In a critically ill patient, the goal of nutritional support is to supply energy and protein load adequate to their metabolic and nutritional needs at a given time of disease by enteral and/or parenteral route [1,2]. However, these patients are highly variable in terms of admission motive, comorbidities, phase of the disease, or organ dysfunction. So, in such a heterogeneous population, it would be correct to assume an approach not only of optimal nutrition individualization per patient but also by the evolution of the pathophysiological process [3].

Currently, both the European Society for Clinical Nutrition and Metabolism (ESPEN) and the American Society for Parenteral and Enteral Nutrition (ASPEN) recommend an energy intake of up to 70% of nutritional needs in the first week using hypocaloric nutrition, in the absence of an evaluation of the energy needs by indirect calorimetry [4,5].

This homogeneous approach, for such a heterogeneous population of patients, may not be the most appropriate [6]. In fact, this recommendation has been questioned in recent years by several authors and has been added to the latest guidelines, suggesting not to exceed 70% of the calculated energy expenditure (EE) during the first week [7-9].

Recent studies that compared full feeding to underfeeding were based on predictive equations and had a lack of not only timing definition on evaluation but also initiating nutritional support, thus not taking into account the clinical state of the patient [3].

Currently, the most appropriate prescription of sedation and analgesia, as well as more aggressive and early control of temperature and multiorgan failure, has allowed attenuating hypermetabolic and catabolic states, common in critically ill patients [10-12]. So, caloric evaluation should come in line with the improvement of clinical status due to medical care provided to the critically ill and consequently the adequacy of nutritional support in line with hypocaloric nutrition [13].

Therefore, it is relevant to determine the minimum and maximum energy intake to be provided at the different stages of illness, in order to minimize the risks associated with under- or overfeeding and optimize the nutritional support.

The best tool for assessing the nutritional needs of critically ill patients still remains to be indirect calorimetry [14-18], which calculates energy expenditure through the respiratory quotient (RQ), determined by measuring oxygen consumption and carbon dioxide production, measurable in the respiratory gases inspired and expired by the patient [13]. The TICACOS study, which compared nutritional support according to the aforementioned guidelines with nutritional support, adapted to energy expenditure (EE), calculated by indirect calorimetry, concluded that the second approach resulted in reduced morbidity and mortality [19,20].

The present study intends to compare the energy needs using simple weight-based equations (20-25 kcal/kg/day) [4,5] calculated by the Harris-Benedict equation [21] with indirect calorimetry in the first week of hospitalization in the intensive care unit (ICU) and evaluate possible stress factors influencing energy expenditure.

## Materials and methods

A prospective observational study was carried out in the last four months of 2019, at the polyvalent ICU of Centro Hospitalar Universitário do Porto (CHUP), with a single moment of data collection during the first seven days of hospitalization, without any change in the normal functioning of the ICU or in the therapy applied to the patients. The following data was collected: age, sex, actual weight (in obese patients, the real weight was used), height, in-hospital outcome, in-hospital mortality, diagnosis by group (medical or surgical; due to the low number of patients in the study, the trauma patients were included in the surgical group for statistical analysis purposes), and severity index by application of the sequential organ failure assessment (SOFA) score on the day of measurement by indirect calorimetry. Other data was also collected to allow for the evaluation of potential stress factors influencing energy expenditure, namely, the use of vasopressors, the Richmond Agitation-Sedation Scale, and body temperature.

The study was authorized by the Board of Directors of CHUP and by the Ethics Committee for Health.

In order to carry out the indirect calorimetry, the Deltatrac^TM^ II device (Datex-Ohmeda Inc., Madison, WI) was used, performing a single measurement, with a minimum time of 120 minutes, during the first week of hospitalization. The calorimeter was calibrated (gas and pressure), before each measurement, with the gas mixture indicated by the device manufacturer (95% O_2_ and 5% CO_2_).

To assess the severity of organ dysfunction, the SOFA severity index was used, which makes it possible to assess the function of six organs (the nervous, cardiovascular, respiratory, hematological, hepatic, and renal systems) and estimate the risk of morbidity and mortality of critically ill patients. Patients with a score equal to or greater than 9 are considered to have a higher mortality rate, due to the greater severity of multiorgan dysfunction [22]. It was considered that all patients with a body temperature greater than 37.5°C (tympanic) would have fever [23]. All patients who were medicated with propofol or midazolam were considered to be sedated.

To estimate the energy needs, the Harris-Benedict predictive equation was applied [20], considering, as a good estimate of energy expenditure, the values calculated by Harris-Benedict that did not vary by more than 10% in relation to the value actually measured in indirect calorimetry or that did not vary by more than 250 kcal from the actual value [24].

The sample was selected among hospitalized and mechanically ventilated patients, with the exclusion of patients whose expected hospitalization time was less than five days, as well as those under 18 years of age. Patients with respiratory quotient (RQ) values below 0.67 [13] were also excluded, as well as patients with fraction of inspired oxygen (FiO_2_) values above 60% [25].

Data processing for statistical analysis was carried out using the Statistical Package for Social Sciences (SPSS) version 24 statistical program (IBM SPSS Statistics, Armonk, NY). Descriptive statistics were performed, with an evaluation of the normality of continuous variables using the Kolmogorov-Smirnov test, with subsequent calculations of several Student's t-test, Pearson's correlation, root mean square deviation (RMSD), and chi-square test to compare the RMSD value with 250 kcal. Statistical significance was considered with p < 0.05 and a 95% confidence interval.

## Results

The 46 ICU patients had an average age of 64.80 ± 14.68 years old, predominantly male sex (71.7%), with a mortality rate of 21.7% (Table 1). Accordingly, with the classification of the World Health Organization [26], two patients (4.35%) were underweight (BMI < 18.5); 15 (32.61%) had excessive weight; and regarding obesity, two patients were, respectively, in classes II and III, with eight patients (17.40%) in class I of obesity classification. Nineteen patients (41.30%) had a normal BMI (Table 1).

**Table 1:** Population characterization. SD: standard deviation

Number of patients (N)	46
Age, years (average ± SD)	64.80 ± 14.68
Gender: male, n (%)	33 (71.7)
BMI, kg/m^2^ (average ± SD)	26.05 ± 5.50
Outcome on discharge, n (%): improved/dead	36 (78.3)/10 (21.7)
Type of admission, n (%): medical/surgical	16 (34.8)/30 (65.2)
Use of vasopressors, n (%)	16 (34.8)
Sedation, n (%)	29 (63)
Temperature, n (%): febrile/apyretic	25 (54.3)/21 (45.7)
SOFA score (average ± SD)	5.70 ± 3.10

All patients were on invasive ventilation. No patient was considered on palliative care. Most of the patients had a surgical admission (65.2%), were sedated (63.0%) and febrile (54.3%), and were not on vasopressor support (65.2%). The average SOFA score was 5.70 ± 3.1; 13% (n = 6) had a SOFA score equal to or over 9, while the other 87% (n = 40) had a SOFA score lower than 9.

Regarding outcome, 78.3% of patients had an improved status on discharge from the ICU, with a mortality rate upon discharge from the hospital of 21.7% (n = 10).

In regard to energy consumption per kilogram of body weight, the values measured by indirect calorimetry were 19.22 ± 4.67 (18.7) kcal/kg of weight, per day, and the estimated values by the Harris-Benedict equation were 20.19 ± 2.03 (20.2) kcal/kg weight per day; no statistically significant differences were observed (Table 2).

**Table 2:** The patient's energy expenditure using indirect calorimetry and the Harris-Benedict equation and concordance using the paired sample Student's t-test. SD: standard deviation

Energy expenditure	Average ± SD	P value
Total (kcal/day): indirect calorimetry/Harris-Benedict	1371.09 ± 314.28/1454.69 ± 232.38	0.069
Weight (kcal/kg/day): indirect calorimetry/Harris-Benedict	19.22 ± 4.67/20.19 ± 2.03	0.140

An analysis of the concordance rate between the energy needs obtained by calorimetry and the Harris-Benedict predictive equation for each patient was carried out through the intraclass correlation coefficient and Bland-Altman analysis. Concerning the intraclass correlation coefficient, it is concluded that there is a weak correlation between energy consumption and the Harris-Benedict predictive equation (Table 2).

**Figure 1:**
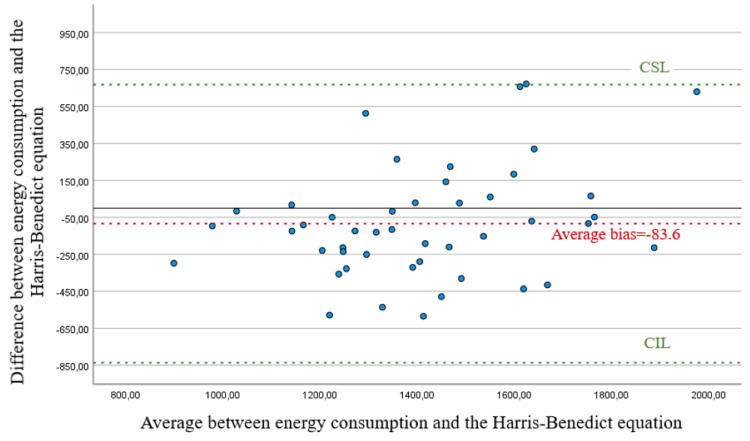
Scatter plot for the difference and average between energy consumption and the Harris-Benedict equation. CSL, concordance superior limit; CIL, concordance inferior limit

**Figure 2:**
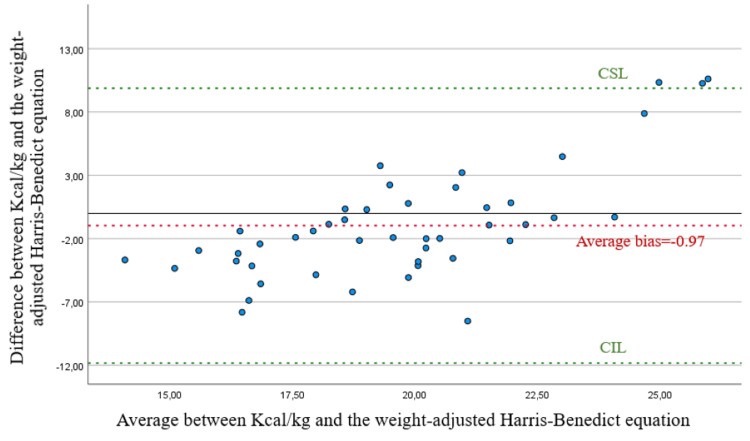
Scatter plot for the difference and average between kcal/kg and the weight-adjusted Harris-Benedict equation. CSL, concordance superior limit; CIL, concordance inferior limit

Applying the Bland-Altman analysis, there is confirmation of the weak correlation between energy consumption and the Harris-Benedict predictive equation. Regarding energy consumption and the Harris-Benedict equation in this population, there is a very high mean bias, and the points are very far from the zero point (Figures 1-2).

As for energy consumption evaluated in kcal/kg of weight and the Harris-Benedict equation, there is a reduced mean bias; however, we should note that on values below 18 and above 20.5, the correlation is weak. In the range from 18 to 20.5, there is a good correlation regarding the value of 70% of energy requirements suggested on the guidelines, with some values above the superior limit of correlation.

On the subject of outcome and type of admission (medical versus surgical), there are no statistically significant differences regarding energy requirements during the first week of admission (Table 3). Regarding SOFA score, the energy expenditure of patients with a lower than 9 score was 19.5 ± 4.9 kcal/kg/day, while in patients with a 9 or higher score, the energy expenditure was 17.32 ± 1.98 kcal/kg/day (p = 0.069).

**Table 3:** Energy expenditure as a function of variables in kcal/kg/day using Student's t-test of independent variables. SD, standard deviation; SOFA, sequential organ failure assessment

Variables	Average ± SD (kcal/kg)	P value
Gender: male/female	19.52 ± 4.55/18.44 ± 5.05	0.510
Type of admission: medical/surgical	18.98 ± 4.77/19.34 ± 4.69	0.805
Outcome on discharge: improved/dead	19.28 ± 4.46/18.97 ± 5.63	0.873
Use of vasopressors: yes/no	18.93 ± 5.11/19.36 ± 4.50	0.782
Sedation: yes/no	18.64 ± 4.52/20.21 ± 4.89	0.287
Temperature: febrile/apyretic	19.27 ± 5.40/19.14 ± 3.74	0.919
SOFA score: ≥9/<9	17.32 ± 1.98/19.5 ± 4.9	0.069

When comparing the results on our population obtained through indirect calorimetry with the formulas advocated in the guidelines, 63% of analyzed patients had an energy expenditure of less than 20 kcal/kg per day (Table 4).

**Table 4:** Adequacy of energy expenditure obtained by indirect calorimetry to guidelines

Energy expenditure	<20 kcal/kg/day	20-25 kcal/kg/day	>25 kcal/kg/day
%	63%	26%	11%

## Discussion

In this study, the energy requirements in 63% of patients assessed by indirect calorimetry were lower than 20 kcal/kg of body weight per day, as estimated by the chosen predictive equation (Harrison-Benedict) (Table 4).

The critically ill patient was classically assumed to be hypermetabolic and hypercatabolic and consequently had, from an early stage of the disease, a higher energy need, which was reflected in guidelines published by different international societies [4,5]. However, nowadays, there is the importance given to the optimization of nutritional support, through evolving the tools of assessment and thus individualizing caloric intake [3,10-12].

As has already been widely discussed, currently, there are several studies [7-9] showing that the nutritional needs of these patients are lower than previously recommended, and the latest ASPEN and ESPEN guidelines advise that in the first week of hospitalization, the critical care patient should be on permissive hypocaloric nutrition, with a goal below 70% of the recommended value [4,5].

Regarding the critically ill patient, we must also take into account, in order to further individualize adequate nutritional support, that this is a patient with a prolonged hospital stay, who has a serious clinical condition with a high mortality rate, which in most cases is often unstable for a period longer than 48 hours, and has a tissue oxygen deficit that limits the metabolism of the provided macronutrients [26,27].

On the subject of the type of admission (medical versus surgical), we have found no statistically significant differences regarding energy requirements; however, our study had a small sample size [28].

However, as shown in our study, the assumed stress factors did not have a significant impact on the increase in the nutritional needs of patients. This is due to the fact that most of these factors considered hypermetabolic and hypercatabolic have been annulled by effective sedation and analgesia and by a better therapeutic approach to our patients [10-12].

This study corroborates the latest changes in guidelines in light of the evolution of the therapeutic approach to patients. By perpetuating a value of caloric need over the last few decades, without adjusting to the reality of the current critical patient and to the evolution of health care, we run the risk of advocating overnutrition for these patients, thus aggravating their clinical condition and outcome.

On the other hand, we found that the most severely ill patients, those with a SOFA score greater than or equal to 9, had lower energy requirements, with this study presenting values that are close to statistical significance (p = 0.069). These patients are mostly hemodynamically unstable, with limited use of dietary macronutrients, so an excessive energy supply will necessarily worsen their clinical condition. In view of these results, the need to optimize nutritional support is evident, particularly in the first week of hospitalization, when they are in a more critical phase clinically.

In addition to indirect calorimetry, the other methods commonly used and evaluated in our study (absolute values provided by the guidelines and values obtained by the Harris-Benedict equation) did not prove to be a useful tool to estimate the actual energy needs of patients. When the Harris-Benedict formula was used, without stress factors, in 67% of patients, the discrepancy between the values calculated by this method and those obtained by indirect calorimetry differed by more than 10%. Similar results were obtained when using the values recommended by the guidelines.

Consequently, there is now a need of revision on the evolution of therapy in critically ill patients, since various stress factors have evolved and are now optimized, leading to an error in the perpetuation of higher values of calorie intake in critically ill patients. The fact that studies carried out by indirect calorimetry, the gold standard for assessing the nutritional needs of these patients, were carried out more than a decade ago and the use of these devices in current clinical practice was discontinued could be the basis for the perpetuation of this error.

The results obtained in this study seem to reinforce the recommendations of several more recent studies that advocate that in the first week of hospitalization, around 60%-70% of caloric needs should be provided, as referred to in the latest ESPEN guidelines [4]. From our point of view, this approach not only should not be considered as permissive underfeeding but also may even constitute adequate nutritional support for the clinical status of the critically ill patient, in the first week of hospitalization.

The limitations of this study are the fact that this is a data collection from a monocentric, heterogeneous, and small sample of patients, which limits the generalization of conclusions for other intensive care units.

## Conclusions

The energy requirements on the first week of hospitalization in critically ill patients, measured by indirect calorimetry, are lower than those calculated by the Harris-Benedict predictive equation and are less than 20 kcal/kg/day in more than half of the cases. Therefore, a goal of 60%-70% of the needs must be supplied when predictive equations are used, as mentioned in the latest ESPEN guidelines.

The severity of the patient may be a decisive factor in relation to the calculation of energy needs in the first week, and it is important that prospective studies be carried out with a larger number of patients.

## References

[1] Prin M, Wunsch H (2012). International comparisons of intensive care: informing outcomes and improving standards. Curr Opin Crit Care.

[2] Martin CM, Hill AD, Burns K, Chen LM (2005). Characteristics and outcomes for critically ill patients with prolonged intensive care unit stays. Crit Care Med.

[3] Singer P, Hiesmayr M, Biolo G (2014). Pragmatic approach to nutrition in the ICU: expert opinion regarding which calorie protein target. Clin Nutr.

[4] Singer P, Blaser AR, Berger MM (2019). ESPEN guideline on clinical nutrition in the intensive care unit. Clin Nutr.

[5] McClave SA, Taylor BE, Martindale RG (2016). Guidelines for the provision and assessment of nutrition support therapy in the adult critically ill patient: Society of Critical Care Medicine (SCCM) and American Society for Parenteral and Enteral Nutrition (A.S.P.E.N.). JPEN J Parenter Enteral Nutr.

[6] Singer P, Cohen J (2016). How could we make nutrition in the intensive care unit simple?. Rev Bras Ter Intensiva.

[7] Rice TW, Wheeler AP, Thompson BT (2012). Initial trophic vs full enteral feeding in patients with acute lung injury: the EDEN randomized trial. JAMA.

[8] Arabi YM, Aldawood AS, Solaiman O (2015). Permissive underfeeding or standard enteral feeding in critical illness. N Engl J Med.

[9] Ibrahim EH, Mehringer L, Prentice D, Sherman G, Schaiff R, Fraser V, Kollef MH (2002). Early versus late enteral feeding of mechanically ventilated patients: results of a clinical trial. JPEN J Parenter Enteral Nutr.

[10] Dickerson RN, Gervasio JM, Riley ML, Murrell JE, Hickerson WL, Kudsk KA, Brown RO (2002). Accuracy of predictive methods to estimate resting energy expenditure of thermally-injured patients. JPEN J Parenter Enteral Nutr.

[11] Van Zanten AR (2015). Full or hypocaloric nutritional support for the critically ill patient: is less really more?. J Thorac Dis.

[12] Pi-Sunyer FX (2000). Overnutrition and undernutrition as modifiers of metabolic processes in disease states. Am J Clin Nutr.

[13] Holdy KE (2004). Monitoring energy metabolism with indirect calorimetry: instruments, interpretation, and clinical application. Nutr Clin Pract.

[14] Ahrens CL, Barletta JF, Kanji S, Tyburski JG, Wilson RF, Janisse JJ, Devlin JW (2005). Effect of low-calorie parenteral nutrition on the incidence and severity of hyperglycemia in surgical patients: a randomized, controlled trial. Crit Care Med.

[15] Stuani Franzosi O, Delfino von Frankenberg A, Loss SH, Silva Leite Nunes D, Rios Vieira SR (2017). Underfeeding versus full enteral feeding in critically ill patients with acute respiratory failure: a systematic review with meta-analysis of randomized controlled trials. Nutr Hosp.

[16] Villet S, Chiolero RL, Bollmann MD, Revelly JP, Cayeux R N MC, Delarue J, Berger MM (2005). Negative impact of hypocaloric feeding and energy balance on clinical outcome in ICU patients. Clin Nutr.

[17] McClave SA, Snider HL (1992). Use of indirect calorimetry in clinical nutrition. Nutr Clin Pract.

[18] Flancbaum L, Choban PS, Sambucco S, Verducci J, Burge JC (1999). Comparison of indirect calorimetry, the Fick method, and prediction equations in estimating the energy requirements of critically ill patients. Am J Clin Nutr.

[19] Singer P, Anbar R, Cohen J (2011). The tight calorie control study (TICACOS): a prospective, randomized, controlled pilot study of nutritional support in critically ill patients. Intensive Care Med.

[20] Douglas CC, Lawrence JC, Bush NC, Oster RA, Gower BA, Darnell BE (2007). Ability of the Harris Benedict formula to predict energy requirements differs with weight history and ethnicity. Nutr Res.

[21] Bendavid I, Lobo DN, Barazzoni R (2021). The centenary of the Harris-Benedict equations: how to assess energy requirements best? Recommendations from the ESPEN expert group. Clin Nutr.

[22] Vincent JL, de Mendonça A, Cantraine F (1998). Use of the SOFA score to assess the incidence of organ dysfunction/failure in intensive care units: results of a multicenter, prospective study. Working group on "sepsis-related problems" of the European Society of Intensive Care Medicine. Crit Care Med.

[23] Dinarello CA, Porat R (2015). Fever. Harrison’s principles of internal medicine.

[24] Boullata J, Williams J, Cottrell F, Hudson L, Compher C (2007). Accurate determination of energy needs in hospitalized patients. J Am Diet Assoc.

[25] Rhodes A (2007). Procedures, techniques, and minimally invasive monitoring in intensive care medicine, fourth edition. Crit Care.

[26] (2000). Obesity: preventing and managing the global epidemic. Report of a WHO consultation. World Health Organ Tech Rep Ser.

[27] Ponce P, Mendes J (2015). Intensive care medicine manual (Article in Portuguese). Lisboa.

[28] Zusman O, Kagan I, Bendavid I, Theilla M, Cohen J, Singer P (2019). Predictive equations versus measured energy expenditure by indirect calorimetry: a retrospective validation. Clin Nutr.

